# Assessing Risk of Progression in Barrett's Esophagus Using a Mass-Spectrometry-Based Proteomic Panel

**DOI:** 10.14309/ctg.0000000000000939

**Published:** 2025-10-24

**Authors:** Andrew Cannon, Rofyda Elhalaby, Igor Ban, Sheeno Thyparambil, Joe Abdo, Catherine E. Hagen, Christopher P. Hartley

**Affiliations:** 1 Department of Laboratory Medicine and Pathology, Mayo Clinic, Rochester, Minnesota, USA;; 2 ProPhase Labs, Garden City, New York, USA;; 3 mProbe, Rockville, Maryland, USA;; 4 Stella Diagnostics, Salt Lake City, Utah, USA.

**Keywords:** Barrett's esophagus, progression, mass spectrometry, proteomics, risk stratification

## Abstract

**INTRODUCTION:**

Esophageal adenocarcinoma (EAC) is an aggressive cancer with poor prognosis. Barrett's esophagus (BE) is a critical precursor of EAC. Patients with BE undergo endoscopic surveillance to monitor disease progression although only a small fraction develop EAC. These procedures are invasive and have limited accuracy in predicting BE progression. We evaluated the utility of an 8-protein mass spectrometry panel in predicting progression in patients with BE.

**METHODS:**

Eighty untreated controls and 20 cases were selected from our institutional tissue registry. Quantitative mass-spectrometry was performed on microdissected tissue sections. Data were split into 80% training and 20% test sets. We used Least Absolute Shrinkage and Selection Operator-regularized regression to train a logistic classifier on training data. Classifier performance was evaluated in test data.

**RESULTS:**

Ninety-two samples had sufficient tissue for mass spectrometry analysis (18 progressors, 74 nonprogressors). The multivariable regression model produced a sensitivity of 100% and a specificity of 39% in the overall cohort, with AUCs of 0.75 and 0.89 in the overall and test cohorts, respectively. Cox proportional hazards time-to-progression (TTP) showed a hazard ratio of 66.1 (95% CI 7.79–561, *P* = 0.00012) for the model prediction.

**DISCUSSION:**

The promising performance of the model generated here suggests that the test may aid in selecting patients most likely to benefit from active BE surveillance. Moreover, the association of this model's prediction with time-to-progression may offer decision support for management of patients likely to progress quickly. These results support continued development of this proteomic panel as a risk stratification tool for patients with BE.

## INTRODUCTION

Esophageal adenocarcinoma (EAC) is an aggressive malignancy with a dismal prognosis (1). Esophageal intestinal metaplasia (Barrett's esophagus, BE), resulting from gastroesophageal reflux disease (GERD), is a well-established precursor of EAC. Although only a small subset of patients with GERD or BE progress to EAC (2,3), the aggressive nature of EAC necessitates endoscopic surveillance of patients diagnosed with BE (4). Traditional surveillance methods, such as endoscopy with biopsy, are limited in their ability to predict progression to EAC. In a meta-analysis of 24 studies encompassing 2,964 patients, the annual risk of progression to high-grade dysplasia or EAC was only 1.7% among patients with BE with low-grade (LG) dysplasia (5). Furthermore, this annual risk varied with the incidence of LG reported in individual studies, suggesting that pathologist variability in diagnosing LG limits the value of histology in risk assessment (5). Similarly, a progression rate of 1.24 cases per 100 person-years was noted for BE with atypia indefinite for dysplasia (IND) (6). As such, most patients undergo endoscopic surveillance without benefit, reducing the cost effectiveness of BE surveillance and exposing millions of patients to undue risks (7,8). Despite widespread and expensive BE surveillance, there remains a critical gap in identifying which patients with BE will progress to EAC.

Currently, 2 distinct approaches are under development to increase the efficacy and safety of BE surveillance. The first approach is less invasive and less costly: nonendoscopic sponge samples are combined with molecular or immunostaining analyses. These methods have been shown to distinguish BE and high-grade dysplasia/EAC (9–11). Although this approach reduces the invasiveness, risk, and cost compared with traditional endoscopic surveillance, it has limitations in predicting progression risk in early-stage BE and allows for limited concurrent cytologic evaluation.

The second approach uses traditional endoscopic biopsies combined with molecular or proteomic analyses to create risk models. Although this method adds the cost of molecular testing to expensive endoscopies, it offers significant advantages for patient selection: the sophisticated ancillary testing can be integrated with routine combined endoscopic and histopathologic assessment. This permits more precise risk stratification in early-stage BE. For example, short segment BE is common in early disease and is best seen and sampled endoscopically, for subsequent confirmation by routine histology.

Regardless of method, there is an urgent need for biomarkers capable of optimizing surveillance schedules. In this context, proteomic analysis by mass spectrometry shows promise, enabling quantitative multiplex analysis of proteins (up to thousands at a time) associated with disease progression.

Recently, a proteomic panel was developed based on aberrant protein expression in EAC compared with BE (12). The aim of this study was to evaluate this 8-protein mass spectrometry panel (BE-Smart, ProPhase Labs, Garden City, NY) for predicting progression of BE to EAC, with a focus on early BE risk stratification. Such a tool could reduce the incidence and mortality of EAC through targeted monitoring, and decrease the number of required endoscopies, reducing healthcare costs.

## MATERIALS AND METHODS

This study was reviewed and approved by the institutional review board. All patients consented to use of tissue, and the study was conducted in accordance with the Declaration of Helsinki.

We searched our institutional tissue registry between 2005 and 2015 for cases in which the terms “Specialized Barrett's mucosa” or “Barrett's” were present in the diagnostic line, yielding over 34,000 specimens. These were algorithmically processed to create a set of binary descriptors for the presence or absence of GERD, nondysplastic Barrett's mucosa (D0), IND, LG, BE with high-grade (HG) dysplasia and EAC. Samples were then grouped by each medical record number within the set to create a complete record of each patient's histologic follow-up of their esophageal disease. More than 4,500 patients had pathology reports referencing BE with more than 1 biopsy during the 10-year period.

**Figure 1.**
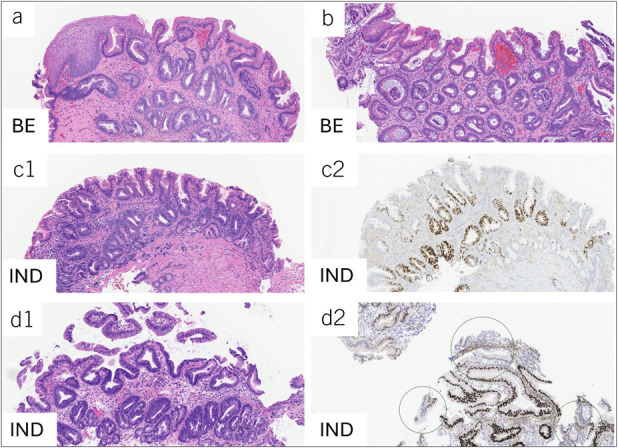
Representative histologic images showing 2 separate examples of nondysplastic Barrett's esophagus (BE). (**a**) and (**b**) Hematoxylin and Eosin, ×100 magnification in contrast to cases diagnosed as Barrett's esophagus with epithelial atypia, IND. The epithelial atypia (nuclear enlargement, hyperchromasia, and stratification) is confined to the epithelial crypts, sometimes referred to as basal crypt atypia (Panels C1 and D1, Hematoxylin and Eosin, ×100 magnification). P53 is overexpressed in the atypical crypts, but there is wild-type expression at the surface of the epithelium. (Panels C2 and D2, P53 immunohistochemistry, ×100 magnification). IND, indefinite for dysplasia.

From this population, 100 formalin-fixed paraffin-embedded tissues were selected for this case-control study (see Supplementary Figure S1, Supplementary Digital Content, http://links.lww.com/CTG/B412). Included cases were selected to be a proportional sampling of the progressor and nonprogressor populations for index histology, age, and sex present in the entire cohort (see Supplementary Table S1, Supplementary Digital Content, http://links.lww.com/CTG/B404). All patients were diagnosed with BE, IND, or LG (Figure 1). Progression was defined as the development of high-grade dysplasia or carcinoma by standard histologic criteria. Only progressors (n = 20) with ≥6 months between diagnosis and progression were included to decrease the likelihood of prevalent HG or EAC. Eighty controls with >5 years of follow-up, median surveillance interval <3 years, and no histologic evidence of HG or EAC were selected. Critically, no controls underwent localized surgical or ablative therapy for BE. Samples were processed using Liquid Tissue (Expression Pathology Inc, Rockville, MD) to allow for proteomic analysis (13). Protein expression was analyzed using triple quadrupole mass spectrometry (QExactive, Thermo Fisher) by mProbe, Inc. (Rockville, MD), a College of American Pathologists accredited, Clinical Laboratory Improvement Amendments certified laboratory. The panel included 13 proteotypic peptides present in 8 unique proteins. Table 1 provides a summary of the target proteins, the proteotypic peptides used in mass spectrometry, and the general pattern of expression in EAC compared with nondysplastic BE (12).

**Table 1.** Summary of protein targets, the proteotypic peptides used in mass spectrometry analysis and the relative expression of the protein in esophageal adenocarcinoma compared with nondysplastic BE (12)

Protein	Proteotypic peptide	Relative expression in esophageal adenocarcinoma
CNDP2	LP	Underexpressed
	TVF	
DAD1	FLE	Similar
	ADF	
GPI	LQQ	Overexpressed
IGS15	IGV	Similar
	LAV	
LTF	DGA	Similar
	FQL	
S100p	YSG	Underexpressed
	ELP	
SET	LNE	Overexpressed
UBE2N	YFH	Overexpressed

ADF, alanine-aspartic acid-phenylalanine; DGA, aspartic acid-glycine-alanine; ELP, proline-glutamic acid-leucine; FLE, phenylalanine-leucine-glutamic acid; FQL, phenylalanine glutamine leucine; GPI, glucose-6-phosphate isomerase; IGV, isoleucine glycine valine; LAV, leucine alanine valine; LNE, leucine-asparagine-glutamic acid; LP, leucine proline; LQQ, leucine glutamine; LTF, lactoferrin; TVF, threonine-valine-phenylalanine; YFH, tyrosine phenylalanine histidine; YSG, tyrosine serine glycine.

### Statistical analysis

Statistical analyses were conducted in R version 4.2.1. The distribution of cases across grades of dysplasia in BE was analyzed using a Fisher exact test. Protein expression data were log-normalized, and the cohort was divided into random 80% training and 20% test sets. Three Least Absolute Shrinkage and Selection Operator (LASSO)-regularized logistic regression model was optimized and trained on the training set using the caret package. One model (base model) included only clinicopathologic data including, age, sex, histologic diagnosis, and BE segment length. The second model included only features from protein quantification by mass spectrometry (protein model). The final model includes both protein and clinicopathologic features (full model). Model performance was assessed in the test data, and permutation tests were used to compare model performance to that of an optimized model of randomized protein expression. Univariable and multivariable Cox proportional hazards models, including the protein-based model prediction and clinicopathologic features were used to assess the relationship of model predictions with time to progression (TTP). Wilcoxon tests were used to compare predictions between progressors and nonprogressors across grades of dysplasia in BE. Using a 2-sided alpha = 0.05, n_1_ = 20 progressors, n_2_ = 80 nonprogressors, the minimum area under curve (AUC) detectable with 80% power is approximately 0.75–0.80 which represents moderate to good discriminatory performance (Hanley and McNeil Approximation for Receiver operating characteristic [ROC] AUC power), which we deemed acceptable for a pilot study. Finally, *t*-tests and univariate ROC curves were used to explore the association of individual protein markers with progression.

### Reporting standards and transparency

This study follows key elements of the Standards for Reporting of Diagnostic Accuracy Studies (STARD) 2015 reporting guidelines with a few exceptions owing to the exploratory nature of this study. Mass spectrometry testing was blinded to clinical outcomes, whereas supervised model training required access to progression labels. Regarding indeterminate values, for the 8-protein panel, the LASSO model assigns a binary classification for each case, and as such, there are no indeterminate values. Last, interassay and intra-assay comparisons were not performed as this was the first exploratory evaluation of the 8-protein panel in progressors and nonprogressors. Future validation studies will be necessary. The remainder of the STARD checklist is addressed elsewhere in the article. A comprehensive checklist is included in Supplementary Table S2 (see Supplementary Digital Content, http://links.lww.com/CTG/B404).

## RESULTS

**Figure 2.**
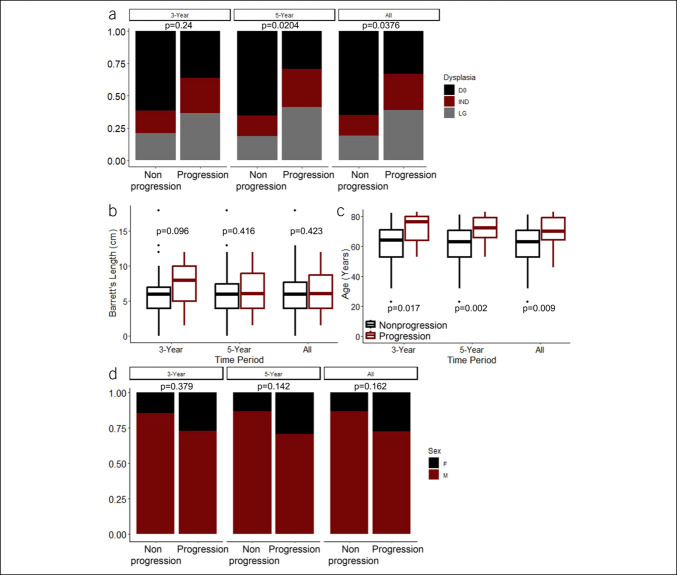
Association of degree of dysplasia (**a**), Barrett’s segment length (**b**), age (**c**), and sex (**d**) with progression status. In each graph, left represents progression status at or before 3 years from the date that the analyzed sample was collected, middle shows progression status at or before 5 years from the date of sample collection, and right depicts the association of the listed feature with progression at any time point after sample collection. *P*-values produced by Fisher exact tests and Mann-Whitney U tests for categorical and numeric variables, respectively. IND, indefinite for dysplasia; LG, low-grade.

Of the 100 samples initially selected, 97 had sufficient material to allow sectioning for research. From these 97 specimens, 92 (95%) had sufficient protein concentration after processing for mass spectrometry analysis. The 92 samples for which mass spectra were successfully obtained were comprised of 18 progressors and 74 nonprogressors, comprising these degrees of BE dysplasia: 54 D0, 17 IND, and 21 LG. The relationship of degree of dysplasia, Barrett's segment length, age, and sex with progression status is demonstrated in Figure 2. The group of patients who progressed before 5 years or at any point after diagnosis had a statistically significant increase in the proportion of IND (29% vs 16% 5-year progression) and LG (41% vs 19% 5-year progression). The same association was not demonstrated between progressors and nonprogressors at or before 3 years of follow-up. In addition, increasing age was significantly associated with progression status at all 3 time points. There was no significant association between Barrett's segment length and sex with progression at any of the time points. Overall, these findings confirm the loose, time-dependent relationship between the presence of dysplasia/epithelial atypia and age with progression. Because the associations of histologic dysplasia and age are significant, they must be accounted for in risk models. However, the inconsistency of this relationship severely limits the utility of these features with respect to predicting BE progression.

**Figure 3.**
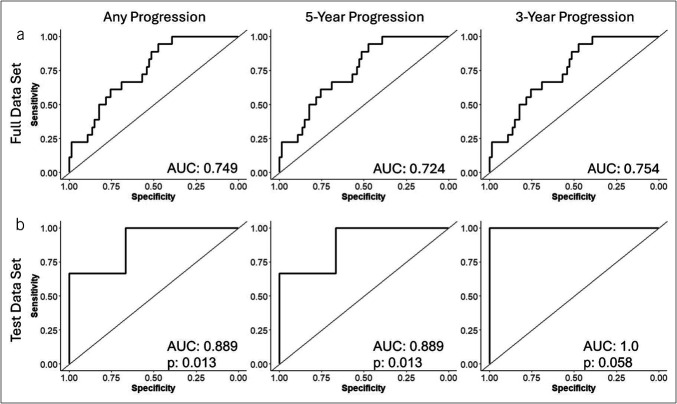
Summary of diagnostic performance of the LASSO-regularized multivariable regression model in (**a**) the full 92-patient cohort and (**b**) the 20% test Data set. Left corresponds to performance with progression status based on any progression after sample collection. Middle shows progression status based on progression at or before 5 years from sample collection and right shows progression at or before 3 years after sample collection. *P*-values generated by permutation test with randomized feature data for training and test set, LASSO-regularized regression model fitting on randomized data and performance tested in the same 20% test set split; the reported *P*-value represents proportion of random models with AUC equal to or exceeding the performance of the real model. AUC, LASSO, Least Absolute Shrinkage and Selection Operator.

We generated a LASSO-regularized logistic regression model using a training Data set comprised a random 80% split of the data. The optimal lambda was selected using 10-fold cross validation to maximize the specificity of the model at 100% sensitivity. This model was subsequently applied to the training data and the maximum cut point to produce 100% sensitivity was selected (Table 2 and Supplemental Data 1 [see Supplementary Digital Content, http://links.lww.com/CTG/B405]). The selected model and cut point were then applied to the 20% test Data set to evaluate performance. ROC curves were used to summarize the predictive performance of the model in the test set and the full Data set (Figure 3). The model demonstrated 100% (95% CI 81.4–100%) sensitivity and specificities of 39.2% (95% CI 28.0–51.2), 38.7%, and 35.8% for any progression, progression at or before 5 years, and progression at or before 3 years, respectively, in the full Data set. The false discovery rate for the full cohort for progression at any time was 71%. Overall, these findings compare favorably with histology alone which produced a sensitivity of 38.9%, a specificity of 81.1%, and a false discovery rate (FDR) of 66.7% when LG was considered a positive result. Critically, the model seemed to perform better in the test Data set with AUCs of 0.89 and 1.0 for any progression/5-year progression and 3-year progression, respectively. In the test cohort, the sensitivity and specificity were 100% and 46.7%, respectively. Despite the small sample size in the test data, this performance was statistically different from randomized models optimized and fit to the data using the same method. A 2 × 2 table summarizes performance in the independent test set (see Supplementary Table S3, Supplementary Digital Content, http://links.lww.com/CTG/B404).

**Table 2.** Summary of L1 LASSO-regularized regression coefficients

	Lambda-regularized Coefficients	Odds ratio
Intercept	−1.017	0.362
CNDP2_LP	−0.87686	0.416
CNDP2_TVF	0	NA
DAD1_FLE	−1.16251	0.313
DAD1_ADF	0	NA
GPI_LQQ	0.798102	2.221
IGS15_IGV	0.10569	1.111
IGS15_LAV	0.075147	1.078
LTF_DGA	0.557412	1.745
LTF_FQL	0.207567	1.231
S100p_YSG	0.455147	1.57
S100p_ELP	−0.17166	0.842
SET_LNE	0	NA
UBE2N_YFH	0.161032	0.851

LASSO, least absolute shrinkage and selection operator.

Lambda was optimized between 0.0017 and 0.1. The Lambda value with the best performance (0.00363) was used for model generation. Note that a coefficient of 0 eliminates the feature from the model. *P*-values are not calculated for coefficients as such values cannot be interpreted accurately following regularization.

We compared the performance of the protein-based model to the performance of 2 additional models to understand the incremental improvement gained by adding the protein features. The first model included only clinical and pathologic features. Although this model performed similarly to the protein-based model in the training data, its performance in test data was comparatively poor (Table 3, see Supplementary Figure S2, Supplementary Digital Content, http://links.lww.com/CTG/B412). In contrast, the model resultant of the combination of the protein and clinicopathologic features (Full model) had improved predictive performance compared with the clinicopathologic model and to a much lesser extent the protein-based model. From this, 2 things are important to note. In the full model, the LASSO selection process retained 12 of 13 peptide markers, representing 7 of 8 proteins, and all clinicopathologic features suggesting that each included feature contributed independently to the predictive performance (Table 3, Supplementary Figure S2 [see Supplementary Digital Content, http://links.lww.com/CTG/B412] and Supplementary Table S4 [see Supplementary Digital Content, http://links.lww.com/CTG/B404]). Second, in the full model, the *P*-values in the test data were not significant despite improved performance; this is likely the result of increased model complexity combined with limited test data.

**Table 3.** Summary of AUC and specificity for 3 predictive models of progression risk in Barrett's esophagus

	Full data		Test data		Full data		Test data	
Model	AUC	*P* value	AUC	*P* value	Specificity	*P* value	Specificity	*P* value
Base	0.732	0.070	0.622	0.341	0.108	0.348	0.20	0.361
Protein	0.749	0.243	0.889	0.013	0.392	0.043	0.467	0.146
Full	0.878	0.021	0.867	0.351	0.351	0.347	0.333	0.718

Base model includes dysplasia, age, sex, and Barrett segment length. The proteome model includes only features derived from the 8 protein mass spectrometry panel. The full model includes the combination clinicopathologic and proteomic features. *P*-values generated by permutation tests including randomized feature space of each respective trained model.

**Figure 4.**
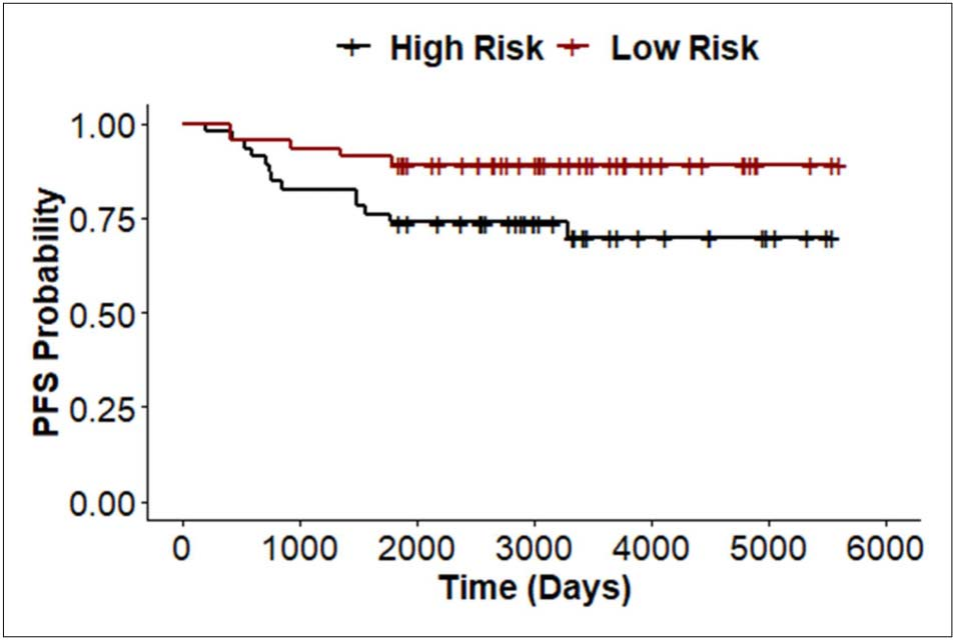
Kaplan-Meier curve of patients stratified by median model risk prediction. Statistics were calculated using the model prediction as a continuous variable using a univariable cox proportional hazards model (*P* = 0.00012, HR 66.12). PFS, progression free survival.

Although the model had promising predictive performance, we questioned if a higher predicted risk of progression by the model correlated with a shorter TTP. Accordingly, we used an univariable Cox proportional hazards model to assess the association of predicted risk of progression with time to progression. Predicted risk of progression was strongly associated with time to progression (hazard ratio [HR] 66.12, 95% CI: 7.79–561, *P* = 0.00012; Figure 4). The association of the predicted risk with time to progression remained highly significant in multivariable models including age, sex, Barrett segment length, and dysplasia (Table 4). This finding indicates that the predicted risk on average was higher in patients that progressed more quickly than those with longer times to progression even after accounting for known patient and disease risk factors.

**Table 4.** Summary of multivariable cox proportional hazards model of time to progression including the proteome model prediction and clinicopathologic characteristics

Feature	Coef	HR	Confidence interval	*P* value
Model prediction	6.752	856.1	41.5–17666.2	1.23 × 10-5
Barrett length	0.102	1.108	0.948–1.294	0.197
Sex (male)	−1.105	0.331	0.097–1.130	0.078
Age	0.060	1.061	1.009–1.116	0.021
Dysplasia (IND)	0.816	2.261	0.612–8.356	0.221
Dysplasia (LG)	1.483	4.406	1.284–15.117	0.0184

IND, indefinite for dysplasia; LG, low-grade.

**Figure 5.**
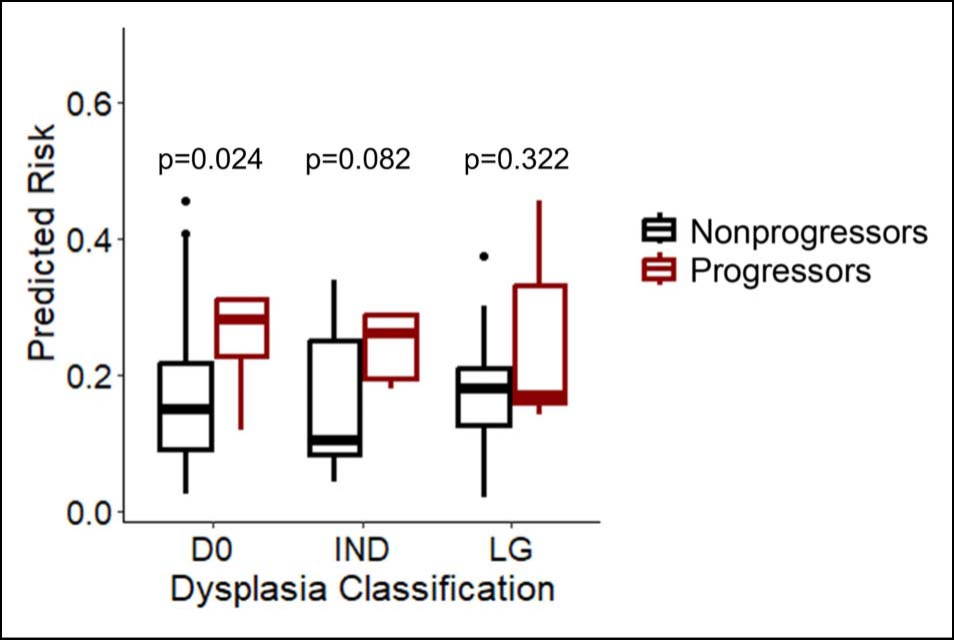
Analysis of model predictions for progressors and nonprogressors across grades of dysplasia at time of index sample. Calculations based on progression after any point of sampling. *P*-values generated by Mann-Whitney U tests. IND, indefinite for dysplasia; LG, low-grade.

Because of the statistical association of histologic dysplasia with progression, we evaluated the predicted risk across grades of dysplasia and progressor status (Figure 5). Surprisingly, the model produced the greatest separation between progressors and nonprogressors in patients with D0 (*P* = 0.024) followed by patients with IND (*P* = 0.082). The model provided the least separation in the cohort of patients with LG. The specificity of the model in the cohort of patients with D0 and IND was 43.3% compared with 21.4% for patients with index biopsies showing LG; the sensitivity for the selected cut point was 100% in all subgroups. Overall, these findings suggest that the model performs best in patients without definitive dysplasia. Consequently, the model prediction provides information that is distinct from histologic dysplasia. Furthermore, the model's prediction may be most informative in specimens that otherwise lack indications of aggressive behavior.

Finally, we explored the associations of individual markers with progression using t-test-based pairwise comparisons of protein expression and univariable ROC curves. Pairwise comparisons of protein expression between progressors and nonprogressors reveal a strong, statistically significant, positive association of lactoferrin (LTF) expression with progression in univariable analysis (Table 5). Critically, this association corresponds to univariable predictive performance (Table 6) and being a correlate of increased risk of progression in the full model.

**Table 5.** T-test results for the comparison of protein marker expression in progressors and nonprogressors

Protein	All		5 yr		3 yr	
	Fold change	*P*-value	Fold change	*P*-value	Fold change	*P*-value
CNDP2_LP	0.864	0.170	0.954	0.512	0.983	0.828
DAD1_FLE	0.960	0.521	0.979	0.744	0.989	0.894
GPI_LQQ	1.056	0.351	1.040	0.507	1.041	0.535
IGS15_IGV	1.085	0.462	1.099	0.412	1.231	0.137
IGS15_LAV	1.058	0.615	1.093	0.439	1.194	0.263
LTF_DGA	**2.039**	**0.008**	**1.985**	**0.014**	**2.577**	**0.014**
LTF_FQL	**2.607**	**0.006**	**2.473**	**0.012**	**2.986**	**0.033**
S100p_ELP	0.935	0.473	0.915	0.350	0.836	0.106
S100p_YSG	0.969	0.755	0.948	0.607	0.847	0.188
UBE2N_YFH	1.072	0.524	1.046	0.685	1.056	0.714

Fold change values are the mean expression of the protein in progressors relative to the mean expression in nonprogressors, such that values less than 1 indicate higher expression in nonprogressors, whereas values ＞1 indicate higher expression in progressors. Bold text highlights results with p-values <0.05.

**Table 6.** Summary of univariable area under the curve statistics for each peptide present in the final model for all progression events, 5-yr progression and 3-yr progression events

Protein	AUC all	AUC 5 yr	AUC 3 yr
CNDP2_LP	0.59	0.44	0.47
DAD1_FLE	0.56	0.53	0.52
GPI_LQQ	0.56	0.54	0.54
IGS15_IGV	0.56	0.57	0.64
IGS15_LAV	0.53	0.55	0.60
LTF_DGA	0.69	0.68	0.75
LTF_FQL	0.72	0.70	0.72
S100p_ELP	0.56	0.58	0.65
S100p_YSG	0.48	0.54	0.64
UBE2N_YFH	0.52	0.50	0.52
Dysplasia	0.66	0.69	0.63

Note the univariable dysplasia model codes dysplasia as an ordinal variable with nondysplastic BE coded as 0, BE with atypia indefinite for dysplasia as 1, and BE with low-grade dysplasia as 2.

## DISCUSSION

We used mass spectrometry to quantify protein markers in samples of BE to generate a model predictive of risk of progression to HG or EAC. In this context, the use of LASSO regression aids in efficient feature selection while also limiting the risk of statistical overfitting. The resulting model identifies at-risk patients with high sensitivity and reasonable specificity. Notably, the AUCs achieved in our test Data set (0.89–1.0 for any progression/5-year progression and 3-year progression, respectively) exceeded the minimum detectable AUC of 0.75–0.80 that our study was powered to detect, suggesting a stronger effect size than anticipated by power analysis. Consequently, the model offers preliminary protein-based insights into which patients are most likely to benefit from close surveillance. Furthermore, subgroup analysis suggests better performance in patients without definitive dysplasia, allowing stratification of patients otherwise deemed to be at minimal risk of progression.

Notably, for the cohort used in this study we excluded patients that progressed in under 6 months from the index biopsy to exclude to the extent possible cases with prevalent dysplasia. This deviates from the currently accepted standard. In our cohort, only 1 patient progressed in less than 1 year, and in this patient's index biopsy they had extensively sampled low-grade dysplasia. We chose to include this patient as.

1. The rate of progression to clinically actionable disease was not excessive.
2. The patient had underlying dysplasia which is known to markedly increase the observed rates of progression in these patients, and
3. Any ancillary test of this nature would ideally highlight patients with prevalent dysplasia and who will develop dysplasia as high-risk.

The model provided prognostic information regarding progression within 3 years, and within 5 years—and in 1 case with progression after 7 years. This time range over which the model seems to function indicates that clinical implementation of such a test would have implications for creating safe and cost-effective surveillance protocols. Despite the wide period over which the model provides predictive information, we were also able to show that higher risk predictions are strongly associated with shorter times to progression. Although the model was not validated as predictive of time-to-progression (TTF), this finding raises the distinct possibility that the quantitative predictions of progression risk rendered by the model may guide management by highlighting patients at risk of imminent progression.

Several other technologies have been developed to predict risk of progression in BE. Although a number of clinical parameters—including age, sex, and medical comorbidities—are associated with risk of progression, more advanced methods have gained increasing interest of late (14). Most notably, the TissueCypher Barrett's Esophagus Assay from Castle Biosciences, Inc. (Friendswood, TX) has demonstrated promising performance. The original design of this assay was composed of 15 features derived from immunofluorescence analysis of 10 protein markers to generate 3 risk categories. In the original study, the model produced an AUC of 0.87 in the entire cohort and 0.80 in an independent validation cohort with a hazard ratio of 14.2 when comparing progression of low and high-risk cohorts (15). More recently, this assay has been validated for nondysplastic BE using 38 cases and 38 controls yielding a sensitivity of 30.4% and specificity of 95% (16). In a separate single-blind comparison, the assay produced a sensitivity of 29% and specificity of 86% (17). Finally, meta-analysis of the studies using the TissueCypher test reported a higher sensitivity of 62% with classification of the intermediate-risk and prevalent dysplasia cases as positive. Unfortunately, the specificity at this cut point was not indicated (18). In patients with BE with low-grade dysplasia, the TissueCypher test showed a sensitivity of 71% and specificity of 78.5% compared with gastrointestinal pathologists who had a 63% sensitivity and 73.5% specificity (19). Cumulatively, these results demonstrate promise for the TissueCypher. Here, we demonstrate a similar degree of performance for the mass spectrometry-based assay, but we note that, although TissueCypher's strength lies in its high specificity, the diagnostic utility of the model we developed lies in its high sensitivity. Accordingly, these 2 assays are likely to be used for different purposes in guiding the management of BE.

Sampling remains a critical issue for ancillary testing in the setting of Barretts esophagus. In the case of TissueCypher, the sensitivity of the assay was improved by analysis of multiple spatially and temporally distinct biopsies. It is entirely possible that the assay and model developed here would benefit from testing in multiple biopsies or additional testing performed at multiple levels through a single biopsy specimen. However, implementation of extensive testing in the clinical setting would come with its own set of challenges. Chiefly these methods would increase the manual, preanalytical input, or the cost of testing due to performing the assay multiple times per case. Ultimately additional technologies are likely needed to surmount the challenges associated with sampling in BE.

The proteins included in the proteomic panel were originally selected based on their aberrant expression in EAC compared with BE (12). The present study demonstrated the utility of these markers in modeling risk of progression in D0, IND, and LG on the foundation of literature supporting a mechanistic role of these proteins in progression of upper gastrointestinal malignancies. For instance, carnosine dipeptidase 2 (CNDP2) encodes a nonspecific dipeptidase. In gastric adenocarcinoma, CNDP2 is downregulated (20). Moreover, forced overexpression of CNDP2 in a gastric carcinoma cell line inhibits cellular proliferation, induces cell cycle arrest, and promotes apoptosis through activation of p38 (20,21). Concordant with this tumor-suppressive effect, the expression level of CNDP2 is inversely correlated with progression risk. In contrast to loss of the tumor-suppressive functions of CNDP2, we note that Glucose-6-phosphate isomerase (GPI) expression is associated with increased risk of progression. Corresponding to this observation in our model, GPI is overexpressed in gastric cancer and high expression levels are associated with advanced stage and poor outcomes (22). Knockdown of GPI in-vitro modulated cancer cell metabolism and inhibited the proliferative and invasive phenotypes of cultured gastric cancer cells (22,23). Similarly, knockdown of S100p in MGC-803 and SGC-7901, 2 gastric cancer cell lines, abrogated the ability of these cells to form colonies through promotion of apoptosis corresponding potentially to a positive association with progression risk in our model (24). SET, a nuclear proto-oncogene involved in chromatin remodeling and transcriptional regulation, showed positive association with progression in our model, consistent with its known oncogenic functions. Finally, in the literature, lactoferrin shows a complex relationship with upper gastrointestinal malignancies. Although the protein itself is underexpressed in gastric and EAC (25), high expression of LTF was observed in metaplastic gastric epithelium, and this expression was associated with the development of adenocarcinoma (26). Our data suggest that LTF is a key positive predictor of BE progression. These findings suggest the potential importance of LTF in the development of carcinoma from upper gastrointestinal metaplasia, warranting further investigation. Overall, these studies support a potential mechanistic role of the markers in our panel in the progression of BE to HG or EAC.

This study is not without limitations. Chiefly, the limited sample size limits the generalizability of these findings. Specifically, the samples in this study were derived from a single institution; as such, the populations present within the test and training cohort may not be entirely reflective of the population in which such a test would be used. The promising performance of this method and associated model in the current Data set certainly warrants further investigation in broader, more diverse populations. Similarly, the limited sample size leaves the possibility that important disease features, particularly those that have yet to be identified, may not be well-represented in the cohort. Although we were careful to select samples that would represent many important clinical and pathologic characteristics of BE, analysis of additional samples would almost certainly aid in identifying key strengths and weaknesses of the current model.

In summary, this pilot study demonstrates promising performance for a mass spectrometry-based predictive model of progression risk in BE. With a sensitivity of 100% (95% CI 81.4%–100%) and specificity over 39% (95% CI 28.0–51.2) in the total Data set, this model may allow for improved selection of candidates for endoscopic surveillance. Critically, the model provides greatest stratification of patients with nondysplastic BE, providing clinically meaningful guidance in this important subset of patients with BE. Furthermore, model predictions were strongly associated with TTP, indicating the potential to identify patients at considerable risk of imminent disease progression. In addition to promising predictive performance, there is ample evidence in the literature to support a mechanistic link between the 8 proteins in the panel and the biology of BE progression.

## CONFLICTS OF INTEREST

**Guarantor of the article:** Christopher P. Hartley, MD.

**Specific author contributions:** A.C.: conception and design of the work, data collection, interpretation of data, statistical analysis, drafting of the manuscript, and critical review of the work. Has approved the final draft submitted for publication. R.E.: data collection, interpretation of data, and critical review of the work. Has approved the final draft submitted for publication. I.B.: data collection and critical review of the work. Has approved the final draft submitted for publication. S.T.: data collection, statistical analysis, critical review of the work. Has approved the final draft submitted for publication. J.A.: conception and design of the work, interpretation of data, drafting of the manuscript, and critical review of the work. Has approved the final draft submitted for publication. C.E.H.: interpretation of data, drafting of the manuscript, and critical review of the work. Has approved the final draft submitted for publication. C.P.H.: conception and design of the work, data collection, interpretation of data, statistical analysis, drafting of the manuscript, and critical review of the work. Has approved the final draft submitted for publication.

**Financial support:** ProPhase Labs paid third party laboratory mProbe to perform mass spectrometry analysis on the specimens. ProPhase Labs owns the assay that was used in the study. No direct funding was provided to the authors.

**Potential competing interests:** I.B. employed by ProPhase Labs, which sponsored the study by paying for mass spectrometry analysis. S.T. was employed by mProbe at the time the mass spectrometry analysis was performed; mProbe was the third party laboratory that was paid by ProPhase Labs to perform the analysis. J.A. is an unpaid clinical advisor to ProPhase Labs. None of the other authors have any relevant conflicts of interest.

**IRB approval statement:** This study was reviewed and approved by the institutional review board at the Mayo Clinic (Rochester, MN). All patients consented to the use of their material for research and the study was conducted in accordance with the Declaration of Helsinki.

**Study Highlights**

**WHAT IS KNOWN**

✓ Barrett's esophagus is a precursor to esophageal adenocarcinoma.
✓ Only a small fraction of Barrett's esophagus patients progresses to cancer.
✓ Current surveillance methods have limited accuracy predicting progression risk.
✓ Endoscopic surveillance is invasive, expensive, and often unnecessary.
✓ Histologic dysplasia alone has limited predictive utility for progression.

**WHAT IS NEW HERE**

✓ Novel mass spectrometry proteomic approach for Barrett's esophagus risk stratification.
✓ Model achieved 100% sensitivity predicting progression from early Barrett's.
✓ Model performed best in patients without definitive dysplasia.
✓ Lactoferrin expression was the strongest individual predictor of progression.

## Supplementary Material

SUPPLEMENTARY MATERIAL
