# Supplementary material for: Assessing Risk of Progression in Barrett's Esophagus Using a Mass-Spectrometry-Based Proteomic Panel
Source: Clin Transl Gastroenterol. 2025 Oct 24;16(12):e00939. doi: 10.14309/ctg.0000000000000939 (PMC12727366; doi:10.14309/ctg.0000000000000939)
Supplement: SUPPLEMENTARY MATERIAL [file ct9-16-e00939-s001.docx]

Table S1. Limited demographic summary of progressors and non-progressors. Statistics calculated with Mann-Whittney U test used for Age and Barrett Length, Fisher’s Exact test for sex.

| **Feature** | **Progressors (n=18)** | **Non-progressors (n=74)** | **p-Value** |
| --- | --- | --- | --- |
| Age (Median) | 70 | 63 | 0.009 |
| Sex (% Female) | 38.9 | 13.5 | 0.02 |
| Barrett Length (cm) | 6.48 | 5.94 | 0.423 |

Table S2. STARD 2015 Checklist

| Section & Topic | No | Item | Reported / Location or Comment |
| --- | --- | --- | --- |
| TITLE OR ABSTRACT | 1 | Identification as a study of diagnostic accuracy using at least one measure of accuracy (such as sensitivity, specificity, predictive values, or AUC) | Yes – Abstract includes AUC, sensitivity, specificity; STARD compliance noted in Methods. |
| ABSTRACT | 2 | Structured summary of study design, methods, results, and conclusions | Yes – Abstract is structured. |
| INTRODUCTION | 3 | Scientific and clinical background, including the intended use and clinical role of the index test | Yes – Introduction. |
| INTRODUCTION | 4 | Study objectives and hypotheses | Yes – Introduction. |
| METHODS – Study design | 5 | Whether data collection was planned before the index test and reference standard were performed (prospective study) or after (retrospective study) | Yes – Retrospective study design described in Methods. |
| METHODS – Participants | 6 | Eligibility criteria | Yes – Methods. |
| METHODS – Participants | 7 | On what basis potentially eligible participants were identified | Yes – Based on progression status from tissue registry and laboratory information system records. |
| METHODS – Participants | 8 | Where and when potentially eligible participants were identified (setting, location and dates) | Yes – Described in Methods. |
| METHODS – Participants | 9 | Whether participants formed a consecutive, random or convenience series | Yes – Convenience cohort described in Methods. |
| METHODS – Test methods | 10a | Index test, in sufficient detail to allow replication | Yes – Mass spectrometry and LASSO modeling described. |
| METHODS – Test methods | 10b | Reference standard, in sufficient detail to allow replication | Yes – Histologic progression status defined as reference. |
| METHODS – Test methods | 11 | Rationale for choosing the reference standard (if alternatives exist) | Yes – Histology is clinical gold standard. |
| METHODS – Test methods | 12a | Definition of and rationale for test positivity cut-offs or result categories of the index test, distinguishing pre-specified from exploratory | Yes – Post hoc cut-off chosen in training set to maximize sensitivity. |
| METHODS – Test methods | 12b | Definition of and rationale for test positivity cut-offs or result categories of the reference standard, distinguishing pre-specified from exploratory | Yes – Reference standard defined as progression to HGD/EAC. |
| METHODS – Test methods | 13a | Whether clinical information and reference standard results were available to the performers/readers of the index test | Partially – MS blinded; model development required outcome access. |
| METHODS – Test methods | 13b | Whether clinical information and index test results were available to the assessors of the reference standard | Yes – Reference standard defined retrospectively. |
| METHODS – Analysis | 14 | Methods for estimating or comparing measures of diagnostic accuracy | Yes – AUC, sensitivity, specificity with 2×2 contingency table. |
| METHODS – Analysis | 15 | How indeterminate index test or reference standard results were handled | Yes – Indeterminate histology handled with follow-up; LASSO model does not output indeterminate results mathematically (continuous output with binary cutpoint) |
| METHODS – Analysis | 16 | How missing data on the index test and reference standard were handled | Yes – No missing data noted. |
| METHODS – Analysis | 17 | Any analyses of variability in diagnostic accuracy, distinguishing pre-specified from exploratory | No – Variability analysis not conducted as the LASSO method represents the first attempt to evaluate the 8-protein panel in progressors versus non-progressors. Acknowledged in methods. |
| METHODS – Analysis | 18 | Intended sample size and how it was determined | Yes – statistical analysis subsection of methods |
| RESULTS – Participants | 19 | Flow of participants, using a diagram | Mentioned – Not shown; referenced as Figure S1. |
| RESULTS – Participants | 20 | Baseline demographic and clinical characteristics of participants | Yes – See Supplementary Table S1. |
| RESULTS – Participants | 21a | Distribution of severity of disease in those with the target condition | Yes – Reported in Results. |
| RESULTS – Participants | 21b | Distribution of alternative diagnoses in those without the target condition | Yes – All non-progressors defined by lack of progression. |
| RESULTS – Participants | 22 | Time interval and any clinical interventions between index test and reference standard | Yes – No interventions occurred during follow-up. |
| RESULTS – Test results | 23 | Cross tabulation of the index test results (or their distribution) by the results of the reference standard | Yes – Supplemental Table S2. |
| RESULTS – Test results | 24 | Estimates of diagnostic accuracy and their precision (such as 95% confidence intervals) | Yes – AUC, sensitivity, specificity reported. |
| RESULTS – Test results | 25 | Any adverse events from performing the index test or the reference standard | No – Not applicable to retrospective MS data. |
| DISCUSSION | 26 | Study limitations, including sources of potential bias, statistical uncertainty, and generalisability | Yes |
| DISCUSSION | 27 | Implications for practice, including the intended use and clinical role of the index test | Yes |
| OTHER INFORMATION | 28 | Registration number and name of registry | No – Not registered; exploratory study. |
| OTHER INFORMATION | 29 | Where the full study protocol can be accessed | No – No public protocol. |
| OTHER INFORMATION | 30 | Sources of funding and other support; role of funders | Yes – Described on title page |

Table S3. 2x2 confuson matrix summarizing the diagnostic perfromance of the LASSO regularized protein-based model in the 20% test set for progression at any time point following the index biopsy.

|  | Progressor (Yes) | Progressor (No) | Total |
| --- | --- | --- | --- |
| Test Positive | 3 (TP) | 7 (FP) | 10 |
| Test Negative | 0 (FN) | 8 (TN) | 8 |
| Total | 3 | 15 | 18 |

Table S4. Summary of L1 LASSO-Regularized regression coefficients for the model including protein and clinicopathologic features. Lambda was optimized between 0.0017 and 0.1. The Lambda value with the best performance (0.001784) was used for model generation. Note that a coefficient of 0 eliminates the feature from the model. p-values are not calculated for coefficients as such values cannot be interpreted accurately following regularization.

|  | **Lambda-**  **Regularized**  **Coefficients** | **Odds Ratio** |
| --- | --- | --- |
| **Intercept** | -4.12783 | 0.0161 |
| **CNDP2_LP** | -2.06058 | 0.1274 |
| **CNDP2_TVF** | 0.40202 | 1.4948 |
| **DAD1_FLE** | -2.50830 | 0.0814 |
| **DAD1_ADF** | 0.02500 | 1.0253 |
| **GPI_LQQ** | -0.35318 | 0.7024 |
| **IGS15_IGV** | -0.79372 | 0.4522 |
| **IGS15_LAV** | 2.09268 | 8.1066 |
| **LTF_DGA** | 0.82709 | 2.2867 |
| **LTF_FQL** | 0.26572 | 1.3044 |
| **S100p_YSG** | 4.87247 | 130.64 |
| **S100p_ELP** | -3.24853 | 0.0388 |
| **SET_LNE** | 0.14872 | 1.1603 |
| **UBE2N_YFH** | 0 | NA |
| **Barrett Length** | 0.19023 | 1.2095 |
| **Sex (Male)** | -0.08577 | 0.9178 |
| **Age** | 0.09224 | 1.0966 |
| **Dysplasia (IND)** | 0.93180 | 2.5391 |
| **Dysplasia (LG)** | 1.31919 | 3.7404 |
