# Supplementary material for: Assessing Risk of Progression in Barrett's Esophagus Using a Mass-Spectrometry-Based Proteomic Panel
Source: Clin Transl Gastroenterol. 2025 Oct 24;16(12):e00939. doi: 10.14309/ctg.0000000000000939 (PMC12727366; doi:10.14309/ctg.0000000000000939)
Supplement: SUPPLEMENTARY MATERIAL [file ct9-16-e00939-s003.docx]

Figure S2. Summary of diagnostic performance of the LASSO-regularized multivariable regression models including only clinicopathologic features (**A and B**) and the full model including clinicopathologic and protein features (**C and D**) in the full 92-patient cohort (**A and C**) and the 20% test data set **(B and D).** Left corresponds to performance with progression status based on any progression after sample collection. Middle progression status based on progression at or before five years from sample collection and right progression at or before three years after sample collection. P-values generated by permutation test with randomized feature data for training and test set, LASSO-regularized regression model fitting on randomized data and performance tested in the same 20% test set split; the reported p-value represents proportion of random models with AUC equal to or exceeding the performance of the real model.
